# Human Cytomegalovirus Mediates Unique Monocyte-to-Macrophage Differentiation through the PI3K/SHIP1/Akt Signaling Network

**DOI:** 10.3390/v12060652

**Published:** 2020-06-17

**Authors:** Olesea Cojohari, Jamil Mahmud, Aaron M. Altman, Megan A. Peppenelli, Michael J. Miller, Gary C. Chan

**Affiliations:** Department of Microbiology & Immunology, SUNY Upstate Medical University, Syracuse, NY 13210, USA; Olesea.Cojohari@umassmed.edu (O.C.); MahmudS@upstate.edu (J.M.); AltmanA@upstate.edu (A.M.A.); mpeppenelli@gmail.com (M.A.P.); MilleM@upstate.edu (M.J.M.)

**Keywords:** human cytomegalovirus, monocytes, macrophages, differentiation

## Abstract

Blood monocytes mediate the hematogenous dissemination of human cytomegalovirus (HCMV) in the host. However, monocytes have a short 48-hour (h) lifespan and are not permissive for viral replication. We previously established that HCMV infection drives differentiation of monocytes into long-lived macrophages to mediate viral dissemination, though the mechanism was unclear. Here, we found that HCMV infection promoted monocyte polarization into distinct macrophages by inducing select M1 and M2 differentiation markers and that Akt played a central role in driving differentiation. Akt’s upstream positive regulators, PI3K and SHIP1, facilitated the expression of the M1/M2 differentiation markers with p110δ being the predominant PI3K isoform inducing differentiation. Downstream of Akt, M1/M2 differentiation was mediated by caspase 3, whose activity was tightly regulated by Akt in a temporal manner. Overall, this study highlights that HCMV employs the PI3K/SHIP1/Akt pathway to regulate caspase 3 activity and drive monocyte differentiation into unique macrophages, which is critical for viral dissemination.

## 1. Introduction

In immunocompromised or immunonaive hosts, human cytomegalovirus (HCMV) infection is a major cause of inflammation-based organ diseases due to the systemic spread of the virus [1,2,3,4,5,6,7,8,9]. During viremia, circulating monocytes are the main cell type in the blood carrying HCMV [10,11,12,13]. Monocytes are also the principal infiltrating cell type positive for viral DNA and antigens in the biopsies of infected organs, indicating that monocytes are involved in the hematogenous dissemination of HCMV [12,13,14,15,16,17,18,19]. However, monocytes are short-lived cells with an approximately 48-h lifespan and are not permissive for viral replication [10,11,13,20,21,22,23]. We and others have previously shown that HCMV overcomes these biological obstacles by promoting monocyte survival and by driving them to differentiate into macrophages, which are long-lived cells and are permissive for viral replication [23,24,25,26,27,28,29,30]. HCMV induces monocyte differentiation into an atypical M1 pro-inflammatory-skewed macrophage expressing select M2 anti-inflammatory macrophage features [24,25,31]. The M1 pro-inflammatory macrophage characteristics, such as enhanced expression of adhesion molecules, cell motility, and transendothelial migration likely facilitate the spread of HCMV from the bloodstream into tissues, while the M2 anti-inflammatory features potentially allow the virus to keep antiviral responses at bay [24,25,26,29,31,32,33,34,35,36]. This unusual M1/M2 reprogramming of infected monocytes is a direct consequence of HCMV’s ability to induce the activation of multiple cellular signaling pathways during viral entry [23,28,29,31,35,37].

HCMV infection of monocytes triggers a rapid and sustained activation of Akt, which occurs when viral glycoprotein gB interacts with epidermal growth factor receptor (EGFR) on the surface of monocytes during viral entry [30,32,34,38]. PI3K, the main positive regulator of Akt, is then rapidly activated following virus binding similarly to PI3K activation by growth factor engagement to cognate cell surface receptors. However, in contrast to normal myeloid growth factors, a simultaneous activation of SHIP1 occurs during HCMV binding leading to a noncanonical activation of Akt [30], characterized by an atypical phosphorylation signature. The virus-specific activation of Akt results in the upregulation of a select subset of Akt-dependent prosurvival proteins, including Mcl-1, HSP27, and XIAP to promote the survival of infected monocytes [27,39]. However, the role of Akt and its signaling network in HCMV-driven M1/M2 monocyte-to-macrophage differentiation remains unclear.

HCMV-induced monocyte-to-macrophage differentiation occurs in the absence of viral replication, suggesting that HCMV regulates the process of differentiation by modulating cellular factors [33,38]. Caspases are proteins with documented functions in initiating and executing apoptosis [40]. However, an accumulating body of literature indicates that caspases are also involved in other non-apoptotic processes, including myeloid differentiation [27,41,42,43,44,45,46]. Caspases 2, 3, 8, and 9 are activated in monocytes undergoing differentiation into macrophages [46]. Caspases 3 and 8 have been shown to drive macrophage differentiation of myeloid cells stimulated with macrophage colony stimulating factor (M-CSF) [44,46,47]. Moreover, successive waves of Akt activation were shown to be critical for caspase activation during macrophage differentiation [44]. We recently showed that HCMV initially blocks caspase 3 activation to allow for monocyte survival prior to 48 h [27]. However, after the 48-h viability gate, HCMV induces controlled levels of caspase 3 activity in infected monocytes, which is necessary to mediate monocyte-to-macrophage differentiation [27]. The early blockade of caspase 3 activation is accomplished by HCMV upregulating two downstream targets of Akt, Mcl-1 and HSP27 [27,39,48]. However, the role of Akt in caspase 3 regulation during the later stages of infection and whether caspase 3 is directly involved in mediating the unique M1/M2 differentiation of infected macrophages are unknown.

Here, we report that upon infection in monocytes, HCMV drives their acquisition of a unique macrophage phenotype by upregulating select M1 pro-inflammatory and M2 anti-inflammatory macrophage differentiation markers, consistent with previous transcriptomic studies. We determined that HCMV-induced Akt activity was necessary for the atypical M1/M2 polarization of differentiating monocytes. Mechanistically, we found that PI3K upstream of Akt mediated the differentiation of infected monocytes with the PI3K p110δ isoform being predominantly responsible for driving differentiation. Concomitant signaling from SHIP1 was also required to mediate the distinct M1/M2 differentiation of infected monocytes. Finally, we determined that caspase 3 was the downstream target of Akt responsible for monocyte differentiation. Specifically, caspase 3 activation was tightly controlled by the virus through Akt in a temporal manner, whereby early Akt activation blocked caspase 3 while late Akt activation was necessary for the controlled activation of caspase 3 responsible for monocyte-to-macrophage differentiation. Together, these data demonstrate that the noncanonical activation of Akt stimulates the non-traditional differentiation of HCMV-infected monocytes required for the viral persistence strategy.

## 2. Materials and Methods

### 2.1. Human Peripheral Blood Monocyte Isolation and Culture

Isolation of human peripheral blood monocytes was performed as previously described [30,33,37,38]. Briefly, blood was drawn from random donors by venipuncture, diluted in RPMI 1640 (Lonza, Walkersville, MD, USA), and centrifuged through Histopaque 1077 (Sigma Aldrich, St. Louis, MO, USA) to remove red blood cells and neutrophils. Mononuclear cells were collected and washed with saline to remove the platelets and then separated by centrifugation through a Percoll (GE Healthcare, Wilkes-Barre, PA, USA) gradient (40.48% and 47.7%). More than 95% of isolated peripheral blood mononuclear cells were monocytes as determined by CD14-positive staining [27]. Cells were washed with saline, resuspended in RPMI 1640 supplemented with 1% human AB serum (Sigma Aldrich, St. Louis, MO, USA), and counted. All experiments were performed in 1–2% human serum at 37 °C in a 5% CO_2_ incubator, unless otherwise stated. All experimental protocols in our study were conducted in accordance with the Declaration of Helsinki, as well as SUNY Upstate Medical University Institutional Review Board (IRBNet ID: 262458-15; Approval: 25 May 2020) and Health Insurance Portability and Accountability Act guidelines for the use of human subjects. All subjects gave their informed consent for inclusion before they participated in the study.

For the inhibitor studies, the following reagents were used: MK-2206 2HCL (MK; an Akt inhibitor), BYL-719 (BYL; a p110α inhibitor), TGX-221 (TGX; a p110β inhibitor), and CAL-101 (CAL; a p110δ inhibitor) from Selleckchem (Houston, TX, USA), LY-294002 (LY; a pan-PI3K inhibitor) from Calbiochem (Billerica, MA, USA), 3-α-aminocholestane (3AC; a SHIP1 inhibitor) from Echelon Biosciences (Salt Lake City, UT, USA), z-DEVD-fmk (a caspase 3 inhibitor) from Santa Cruz Biotechnology (Santa Cruz, CA, USA), and z-VAD-fmk (a pan-caspase inhibitor) from Selleckchem (Houston, TX, USA).

### 2.2. Virus Preparation and Infection

**Figure 1:**
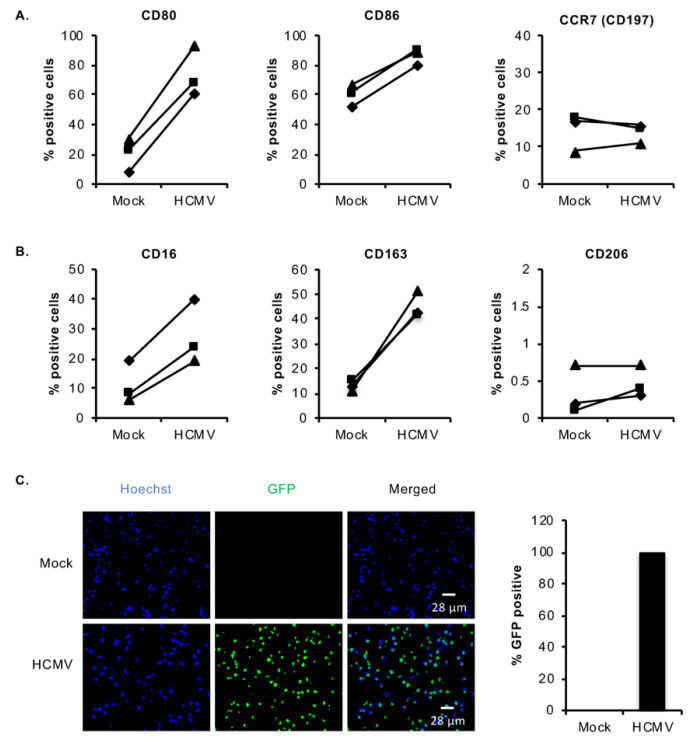
Human cytomegalovirus (HCMV) infection in monocytes induces select M1/M2 macrophage differentiation markers. Human peripheral blood monocytes were mock- or HCMV-infected with TB40E and the percent of cells positive for (**A**) M1 pro-inflammatory macrophage markers or (**B**) M2 anti-inflammatory macrophage markers were measured by flow cytometry at 48 hpi (CD80, CD86, CD16, CD163, and CD206) or 72 hpi (CCR7). (**A**,**B**) Results are representative of 3 independent experiments using monocytes from different donors, with each line representing matched data points from a single donor. (**C**) Monocytes were mock- or HCMV-infected with TB40E-GFP for 72 h. Cells were stained with Hoechst dye and visualized on a ZOE fluorescent cell imager. GFP-positive cells were counted as a percentage of the total number of Hoechst-positive cells. The bar graph represents the mean from 2 independent experiments using monocytes from one donor. Error bars represent the standard deviation.

Low passage (P7–15) human embryonic lung (HEL) 299 fibroblasts (CCL-137, American Type Culture Collection, Manassas, VA, USA) were subcultured in DMEM (Lonza) with 2.5 µg/mL plasmocin (Invivogen, San Diego, CA, USA) and 10% fetal bovine serum (FBS) (Atlanta Biologicals, Flowery Branch, GA, USA). When culture reached confluency, cells were infected with HCMV strain TOWNE/E [49] or strain TB40E [50] without or with GFP under an SV40 promoter [51] in DMEM + 4% FBS. Virus was purified from supernatant on a 20% sorbitol cushion to remove cellular contaminants and resuspended in RPMI 1640. Unless otherwise specified, TB40E and the other strains were used at a multiplicity of infection (MOI) of 5 for each experiment. Greater than 99% of monocytes were infected when an MOI of 5 was used (Figure 1C) [27]. Mock infection was performed by adding an equivalent volume of RPMI 1640 to monocytes.

### 2.3. Western Blot Analysis

Monocytes were harvested in modified RIPA buffer (50 mM Tris-HCl [pH 7.5], 5 mM EDTA, 100 mM NaCl, 1% Triton X-100, 0.1% SDS, 10% Glycerol) supplemented with Protease Inhibitor Cocktail (Sigma) and Phosphatase Inhibitor Cocktails 2 and 3 (Sigma) for 15 min on ice. The lysates were cleared from cell debris by centrifugation at 4 °C (5 min, 21,130 *g*) and stored at −20 °C until further analysis. Protein samples were solubilized in Laemmli SDS-Sample Non-reducing 6× Buffer (Boston Bioproducts, Boston, MA, USA) supplemented with β-mercaptoethanol (Amresco, Solon, OH, USA) by incubating at 100 °C for 10 min. Equal amounts of total protein from each sample were loaded in each well, separated by SDS-polyacrylamide gel electrophoresis, and transferred to polyvinylidene difluoride membranes (Bio-Rad, Hercules, CA, USA). Blots were blocked in 5% bovine serum albumin (BSA) (Fisher Scientific, Waltham, MA) in Tris-buffered saline Tween (TBST) buffer for 1 h at room temperature (RT) and then incubated with primary antibodies in 5% BSA/TBST buffer overnight at 4 °C. Primary antibodies were purchased from the following companies: anti-phospho (p)-Akt (Ser473) and anti-phospho (p)-Akt (Thr308) from Cell Signaling Technology (Danvers, MA, USA), anti-p110δ and anti-caspase-3 from Santa Cruz Biotechnology (Santa Cruz, CA, USA), and anti-beta actin from Abcam (Cambridge, MA, USA). Blots were then washed and incubated with horseradish peroxidase (HRP)-conjugated secondary antibodies from Santa Cruz Biotechnology and Abcam for 15 min on the SNAP i.d. 2.0 Protein Detection System from MilliporeSigma (Burlington, MA, USA). Blots were washed and chemiluminescence was detected using the Amersham ECL Prime Western Blotting Detection Reagent from GE Healthcare (Piscataway, NJ, USA). Images were captured using the Bio-Rad ChemiDoc XRS+ Molecular Imager or ChemiDoc MP Imaging System (Hercules, CA, USA) and ImageLab software.

### 2.4. Flow Cytometry

Monocytes were washed in 1× PBS and incubated in blocking solution consisting of FACS buffer (145 mM NaCl, 8.45 mM Na_2_HPO_4_, 1.83 mM NaH_2_PO_4_, and 0.1% NaN_3_), 5% BSA, and human FcR binding inhibitor (eBioscience, San Diego, CA, USA) for 20 min on ice. After blocking, cells were stained by adding APC anti-human CD14, Alexa Fluor 488 anti-human CD16, PE/Cy7 anti-human CD163, Brilliant Violet 605 anti-human CD86 (BioLegend, San Diego, CA, USA), and APC-R700 anti-human CD80 antibodies (BD Biosciences, San Jose, CA, USA) or isotype controls on ice. Cells were then washed in 1× PBS and analyzed by flow cytometry using an LSRFortessa cell analyzer and BD FACSDiva software (BD Biosciences, Franklin Lakes, NJ, USA).

For viability studies, monocytes were washed after incubation with the APC anti-human CD14 antibody as described above. Cells were then stained with phycoerythrin-annexin V (BD Pharmingen, Franklin Lakes, NJ, USA) and either Sytox Blue dead cell stain (Life Technologies, Carlsbad, CA, USA) or propidium iodide (PI) dead cell stain (Thermo Fisher Scientific, Waltham, MA, USA) to detect dead and dying cells. After staining, cells were analyzed by flow cytometry as described above. Double negative cells represent live cells, whereas double positive and single positive cells represent dead and/or dying cells.

### 2.5. Microscopy

Human peripheral blood monocytes were seeded on a 96-well plate and either mock- or HCMV-infected with TB40E-GFP at a MOI of 5 for 72 h. Cells were stained with the Hoechst 33342 DNA stain (Thermo Fisher Scientific, Waltham, MA, USA) and visualized on a ZOE fluorescent cell imager (Bio-Rad, Hercules, CA, USA). GFP-positive cells were counted as a percentage of the total number of Hoechst-positive cells using the Fiji software.

### 2.6. Statistical Analysis

All experiments were performed independently a minimum of 3 times using primary monocytes isolated from different blood donors, unless otherwise stated. Data sets obtained from primary monocytes inherently have substantial variation due to donor variability. Consequently, data is displayed as matched experimental data points from individual donors in a side-by-side comparison. Displaying a side-by-side comparison allows for consistent trends between the different donors to be identified that may otherwise be missed when presenting only the mean and that may not be statistically significant given the high number of human donors needed to achieve significance on smaller changes.

## 3. Results

### 3.1. HCMV Infection of Monocytes Induces Select M1/M2 Macrophage Differentiation Markers

HCMV infection of monocytes stimulates their differentiation into macrophages, which is vital for viral spread within the host as, unlike monocytes, macrophages are long-lived and permissive for viral replication [24,25,27,33]. Infected monocytes undergoing differentiation display increased granularity, cell spread, and size, as well as enhanced expression of a pan-macrophage cell surface marker, CD71, and an M2 macrophage marker, CD163 [27]. Transcriptome studies revealed a further unusual M1/M2 polarization of infected monocytes where mostly M1 pro-inflammatory transcripts were upregulated, but also select M2 anti-inflammatory transcripts [24,25]. We aimed to further define the aberrant M1/M2 phenotype of differentiating HCMV-infected monocytes by examining a panel of M1 (CD80, CD86, and CD197) and M2 (CD16, CD163, and CD206) macrophage markers [52,53,54,55,56,57]. Primary peripheral blood monocytes were mock- or HCMV-infected for 48 h and examined for changes in expression of differentiation markers by flow cytometric analysis (Figure 1A,B). In accord with our previous study [27], HCMV infection increased the expression of the M2 marker, CD163, as well as an additional M2 marker, CD16 (Figure 1B). Furthermore, consistent with our transcriptome analysis, the virus also induced M1 differentiation markers, CD80 and CD86 (Figure 1A). However, HCMV infection did not upregulate another M1 marker tested, CD197 (also known as CCR7) at 72 h post infection (hpi), or the M2 marker CD206 at 48 hpi (Figure 1A,B), confirming that HCMV infection of monocytes stimulates a unique monocyte-to-macrophage differentiation process by upregulating select M1 and M2 characteristics required for the viral dissemination strategy. In all experiments, we used a multiplicity of infection (MOI) of 5, as we found that >99% of monocytes were infected when this MOI was used (Figure 1C).

### 3.2. HCMV-Induced Akt Mediates Monocyte-to-Macrophage Differentiation

**Figure 2:**
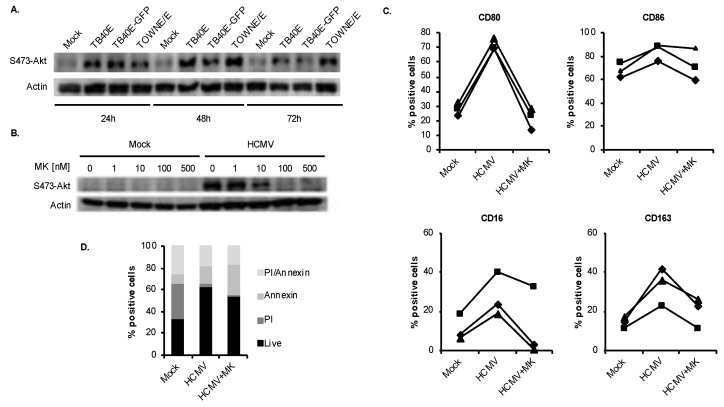
HCMV uses Akt to mediate monocyte-to-macrophage differentiation. (**A**) Human peripheral blood monocytes were mock- or HCMV-infected with TB40E, TB40E-GFP, or TOWNE/E for 24, 48, or 72 h. Levels of p-Akt (S473) and actin (loading control) were detected by immunoblotting from whole cell lysates. (**B**) Monocytes were pretreated for 1 h with an Akt inhibitor, MK-2206 2HCL (MK), at 1, 10, 100, or 500 nM, or vehicle control. Cells were then mock- or HCMV-infected for 24 h and levels of p-Akt and actin detected by immunoblotting from whole cell lysates. (**C**) Monocytes were pretreated for 1 h with 5 μM of MK, or vehicle control, then mock- or HCMV-infected with TB40E for 48 h. The percent of positive cells for M1 (CD80 and CD86) and M2 macrophage markers (CD16 and CD163) were measured by flow cytometry. (**D**) Monocytes were pretreated for 1 h with 5 μM of MK or vehicle control, then mock- or HCMV-infected for 48 h. Viability was measured through propidium iodide (PI) and annexin V staining by flow cytometry. Live cells (double negative for PI and annexin V) are represented by black bars. (**A**–**D**) Results are representative of 3–6 independent experiments using monocytes from different donors.

We next sought to identify the mechanisms by which HCMV regulates the unique M1/M2 polarization of differentiating infected monocytes. Akt activation is critical for monocyte commitment to the macrophage maturation process [58,59,60,61,62] and appears to play an important role in M1/M2 polarization [63,64,65,66,67,68,69,70,71]. We have previously shown that HCMV infection in monocytes activates Akt in a noncanonical manner and maintains its activity through at least 72 hpi [30], suggesting that Akt may be responsible for the HCMV-induced atypical M1/M2 differentiation of infected monocytes. In support of our previous findings with the low passage HCMV TOWNE/E strain [30], clinical HCMV strain TB40E induced the phosphorylation of Akt by 24 hpi, which was maintained at higher levels when compared to mock-infected cells at both 48 h and 72 h (Figure 2A). Note that TB40E-GFP is a GFP-labeled virus [51]. There also appears to be differences in the kinetics of Akt activation induced by the different strains of HCMV. Monocytes infected with the clinical strain (TB40E) reached maximum Akt activation early by 24 hpi while the fibroblast-passaged strain (TOWNE/E) stimulated a more gradual activation, suggesting clinical strains may induce a more rapid activation of Akt. However, more strains need to be tested to confirm this possibility. Nonetheless, our data demonstrate multiple myeloid tropic strains of HCMV stimulate the persistent activation of Akt through the 48-h viability checkpoint. To test the role of Akt in the acquisition of the unique M1/M2 phenotype, we used an Akt inhibitor, MK-2206 (MK). Pretreatment of monocytes with MK prior to infection blocked the ability of HCMV to induce Akt activation in a dose dependent manner (Figure 2B). Inhibition of Akt resulted in a complete ablation of the virus upregulated M1 and M2 differentiation markers as levels were similar to those in mock-infected cells (Figure 2C), indicating that Akt activity is critical for the virally induced M1/M2 monocyte-to-macrophage differentiation process. We verified that the concentration of MK used had minimal cytotoxic effects on monocytes by performing viability studies using propidium iodide, which only enters cells with a compromised cell membrane due to cell death, and annexin V, which is a marker of apoptosis (Figure 2D).

### 3.3. HCMV Predominantly Uses the p110δ Isoform of PI3K to Mediate the M1/M2 Differentiation of Infected Monocytes

**Figure 3:**
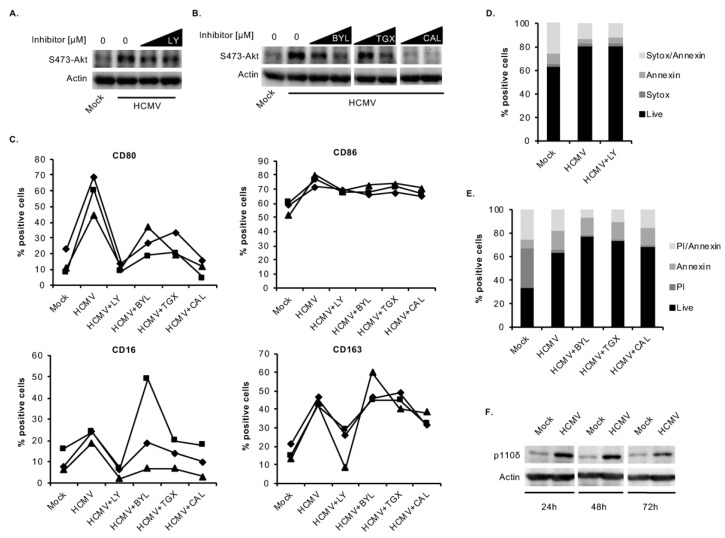
HCMV predominately uses the p110δ isoform of PI3K to mediate monocyte-to-macrophage differentiation. Human peripheral blood monocytes were pretreated for 1 h with vehicle control or (**A**) a pan-PI3K inhibitor (LY) or (**B**) isoform specific inhibitors, BYL for p110α, TGX for p110β, and CAL for p110δ at 0.01 or 0.1 μM. Cells were then mock- or HCMV-infected for 24 h, and levels of p-Akt (S473) and actin (loading control) were detected by immunoblotting from whole cell lysates. (**C**) Monocytes were pretreated for 1 h with vehicle control, LY at 10–25 μM, or BYL, TGX, or CAL at 10 μM. Cells were then mock- or HCMV-infected for 48 h, and the percent of positive cells for M1 (CD80 and CD86) and M2 (CD16 and CD163) macrophage markers was measured by flow cytometry. (**D**) Monocytes were pretreated for 1 h with LY at 25 μM or vehicle control. Cells were then mock- or HCMV-infected for 24 h, and viability was measured by Sytox and annexin V staining. (**E**) Monocytes were pretreated for 1 h with BYL for p110α, TGX, and CAL at 10 μM, or vehicle control. Cells were mock- or HCMV-infected for 48 h, and viability was measured by PI and annexin V staining. (**D**,**E**) Live cells (double negative) are represented by black bars. (**F**) Monocytes were mock- or HCMV-infected for 24, 48, or 72 h. Levels of p110δ and actin were detected by immunoblotting. (**A**–**F**) Results are representative of 3–6 independent experiments using monocytes from different donors.

We and others have previously shown that Akt’s upstream regulator, PI3K, mediates the functional changes associated with HCMV-induced monocyte-to-macrophage differentiation such as enhanced cellular motility, transendothelial migration [34], and upregulation of M1 and M2 transcripts [25], suggesting that PI3K plays a pivotal role in driving the myeloid differentiation process. Accordingly, inhibition of PI3K with a pan inhibitor (LY) abrogated the ability of the virus to induce Akt activation (Figure 3A) and M1/M2 differentiation as expression of CD80, CD86, CD16, and CD163 were reduced (Figure 3C). We confirmed that LY did not exert any cytotoxic effects at the concentrations used (Figure 3D). The catalytic subunit of Class 1A PI3K exists in three isoforms, including p110α, p110β, and p110δ, which have differential roles during M1 and M2 differentiation [66]. We sought to determine the role of the individual PI3K isoforms involved in HCMV-induced differentiation. We used isoform-specific inhibitors and found that, although all three inhibitors reduced HCMV-induced Akt activation (Figure 3B), inhibition with CAL blocked Akt at lower concentrations, suggesting that p110δ may play a more predominant role in regulating Akt-mediated differentiation. Indeed, the p110α (BYL) and p110β (TGX) inhibitors did not reduce or only partially reduced the ability of HCMV to upregulate M1 and M2 markers (Figure 3C). In contrast, the p110δ-specific inhibitor, CAL, reduced the expression of all four differentiation markers to levels approaching those in mock-infected cells similarly to the pan-PI3K inhibitor LY (Figure 3C). These changes are not a result of cytotoxic effects of the inhibitors as concentrations that did not affect cell viability were used (Figure 3E). In further support of p110δ being the main PI3K isoform involved in promoting differentiation of infected monocytes, HCMV stimulated an increase in p110δ expression at 24, 48, and 72 hpi (Figure 3F).

### 3.4. HCMV Engages SHIP1 to Stimulate Monocyte-to-Macrophage Differentiation

**Figure 4:**
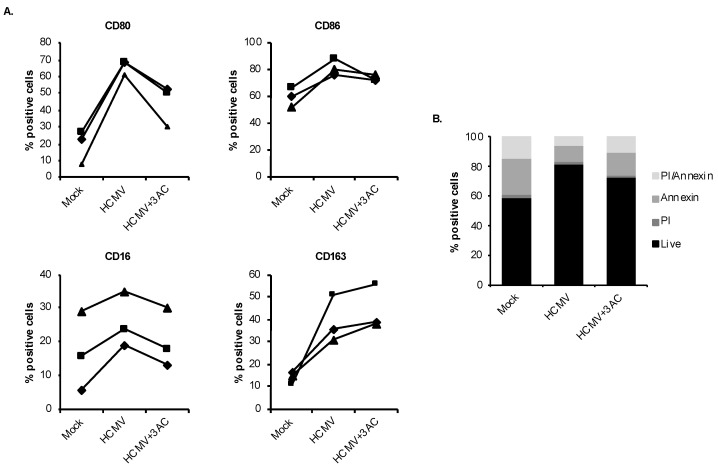
HCMV engages SHIP1 to induce monocyte-to-macrophage differentiation. (**A**) Human peripheral blood monocytes were pretreated for 1 h with a SHIP1 inhibitor (3AC) at 10 μM. Cells were mock- or HCMV-infected for 48 h, and the percent of cells positive for M1 (CD80 and CD86) and M2 (CD16 and CD163) macrophage markers was measured by flow cytometry. (**B**) Monocytes were pretreated for 1 h with 3AC at 10 μM, then mock- or HCMV-infected for 48 h. Viability was measured through PI and annexin V staining by flow cytometry. (**A**,**B**) Results are representative of 3–6 independent experiments using monocytes from different donors.

The subsequent coordinated activation of SHIP1 following PI3K activation by HCMV is responsible for the unique biological activity of Akt following infection of monocytes [30]. Specifically, we recently demonstrated that, in contrast to the established role of SHIP1 as a negative regulator, HCMV uses SHIP1 to stimulate a noncanonical activation of Akt similar to cancer cells [72,73,74] in order to mediate the survival of infected monocytes [30]. Moreover, HCMV infection induces persistent high levels of SHIP1 expression past the 48-h viability checkpoint [30]. Based on these studies, we hypothesized that SHIP1 would also play a role in HCMV-driven monocyte-to-macrophage differentiation. Pretreatment of cells with a SHIP1 specific inhibitor, 3AC, resulted in a robust reduction of virally induced M1 markers CD80 and CD86, as well as the M2 differentiation marker CD16, but had no effect on the M2 marker CD163 (Figure 4A). The concentration of 3AC used in these experiments had minimal effect on cell viability, indicating the effects of 3AC were not from indirect induction of cell death (Figure 4B). Together, these data suggest that HCMV uses coordinated signaling from the PI3K-SHIP1 signaling axis to promote the atypical differentiation of monocytes into M1/M2 macrophages.

### 3.5. HCMV-Induced Caspase 3 Activation after the 48-h Viability Gate Is Required for the Unique M1/M2 Polarization of Infected Monocytes

**Figure 5:**
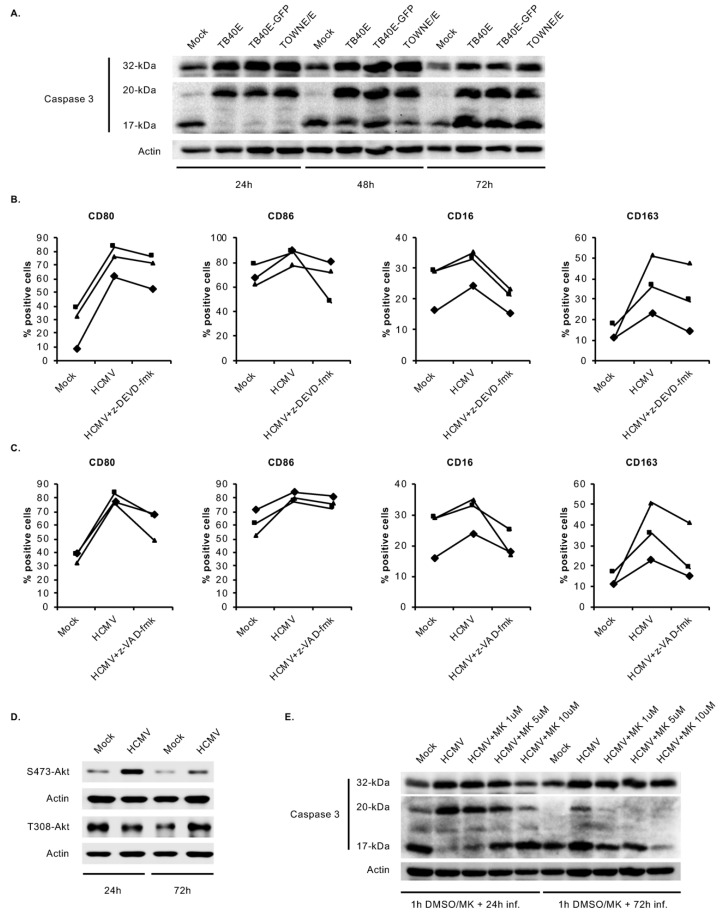
HCMV-induced caspase 3 activation after the 48-h viability gate mediates the unique monocyte-to-macrophage differentiation. (**A**) Human peripheral blood monocytes were mock- or HCMV-infected with TB40E, TB40E-GFP or TOWNE/E for 24, 48, or 72 h. Levels of pro-caspase 3 (32 kDa), intermediate caspase 3 (20 kDa), fully active caspase 3 (17 kDa), and actin were detected by immunoblotting from whole cell lysates. (**B**,**C**) Monocytes were pretreated for 1 h with a vehicle control, (**B**) a caspase 3 inhibitor (z-DEVD-fmk) at 20 μM, or (**C**) a pan-caspase inhibitor (z-VAD-fmk) at 50 μM. Cells were then mock- or HCMV-infected for 48 h and the percent positive cells for M1 (CD80 and CD86) and M2 macrophage markers (CD16 and CD163) were measured by flow cytometry. (**D**) Monocytes were mock- or HCMV-infected for 24 or 72 h. Levels of S473-Akt, T308-Akt, and actin were detected by immunoblotting. (**E**) Monocytes were pretreated for 1 h with MK at 1, 5, or 10 μM, or vehicle control and then mock- or HCMV-infected for 24 h or 72 h. Levels of pro-caspase 3 (32 kDa), intermediate caspase 3 (20 kDa), fully active caspase 3 (17 kDa), and actin were detected by immunoblotting. (**A**–**E**) Results are representative of 3–6 independent experiments using monocytes from different donors.

We have previously shown that HCMV upregulates two antiapoptotic proteins, Mcl-1 and HSP27, in an Akt-dependent manner to cooperatively block caspase 3 activation and the natural progression of early monocyte apoptosis prior to the 48-h viability gate [27,39]. Treatment with high concentrations of PI3K (LY) and Akt (MK) inhibitors, beyond those used in this current study, stimulates caspase 3 activation and apoptosis of infected monocytes, indicating that early caspase 3 activity mediates classical apoptosis [27,38]. Following 48 h, HCMV induces the activation of caspase 3 but at insufficient levels to stimulate apoptosis of infected monocytes [27]. We demonstrated that infected monocytes were unable to differentiate into macrophages if the delayed HCMV-induced activation of caspase 3 was blocked using caspase 3 inhibitors, indicating late caspase 3 activity exhibits an apoptosis-independent function involved in promoting monocyte differentiation [27]. In accord with these findings, we found that infection of monocytes with a clinically derived strain of HCMV induced an early block in caspase 3 activation as fully activated caspase 3 (17 kDa) was absent from infected cells at 24 hpi (Figure 5A). At 48 and 72 hpi, all HCMV strains stimulated a temporal activation of caspase 3, suggesting that a universal activation of caspase 3 after the 48-h viability checkpoint is required for HCMV-induced monocyte-to-macrophage differentiation. To decipher the role of caspase 3 in the unique M1/M2 polarization of infected monocytes, cells were pretreated with a caspase 3 selective inhibitor, z-DEVD-fmk (Figure 5B) then infected with HCMV for 48 h. Inhibition of caspase 3 reduced the expression of M1 markers CD80 and CD86 and the expression of M2 markers CD16 and CD163 (Figure 5B), suggesting caspase 3 is involved in mediating both M1 and M2 differentiation. Although we have previously shown caspase 3 to be solely responsible for mediating the differentiation of HCMV-infected monocytes, other caspases are known to be involved in the differentiation induced by normal myeloid growth factors [27,41,42,43,44,45,46]. Thus, to rule out the involvement of other caspases, we used a pan-caspase inhibitor, z-VAD-fmk, which led to a decrease in M1 and M2 differentiation markers similar to the reduction induced by the caspase 3 selective inhibitor (Figure 5C). These data suggest that caspase 3 is the major caspase regulating HCMV-induced monocyte maturation and polarization.

Finally, we sought to identify the mechanism by which HCMV temporally regulates caspase 3. Akt has been shown to negatively and positively regulate caspase 3 during myeloid differentiation [44,58,69]. Further, we have previously identified several Akt-dependent proteins that controlled caspase 3 activity in HCMV-infected monocytes [27,75]. Based on these observations, we postulated that Akt may be involved in the temporal regulation of caspase 3. The substrate specificity of Akt is dependent on the ratio of serine 473 (S473) and threonine 308 (T308) phosphorylation [76,77,78]; thus, we examined if the Akt phosphorylation profile was different before and after the 48-h viability gate. Consistent with our previous studies, we found HCMV infection led to the site-specific phosphorylation of Akt at S473 but not T308 at 24 hpi (Figure 5D). Interestingly, HCMV stimulated the phosphorylation of Akt at T308 at 72 hpi, suggesting that HCMV-activated Akt may possess distinct biological activities prior to and after the 48-viability checkpoint. Inhibition of Akt prevented the HCMV-induced blockade of caspase 3 activation at 24 hpi (Figure 5E), indicating Akt negatively controls caspase 3 prior to the 48-h viability gate. The negative regulation of caspase 3 by Akt is consistent with our previous findings that Akt is rapidly activated by HCMV to induce survival [30], as well as other studies demonstrating myeloid growth factors induce Akt to block caspase 3 activation and prevent monocyte apoptosis [58,69]. Nevertheless, pretreatment with the Akt inhibitor prevented the delayed activation of caspase 3 at 72 hpi (Figure 5E), suggesting that Akt acts as a positive regulator of caspase 3 after the 48-h viability checkpoint in order to drive M1/M2 differentiation. Though Akt is generally thought of as a negative regulator of caspases, positive regulation of caspase 3 by Akt has also been observed during myeloid growth factor-induced monocyte differentiation [44]. Overall, our data suggest HCMV temporally controls the functional output of Akt activity during the monocyte differentiation process in order to concomitantly drive survival and the distinct M1/M2 differentiation of infected monocytes. To the best of our knowledge, this study is the first to demonstrate that Akt can have simultaneous juxtaposing effects on caspase 3 during myeloid differentiation.

## 4. Discussion

HCMV infection drives monocyte-to-macrophage differentiation, which is critical for HCMV dissemination as macrophages, unlike monocytes, are permissive for viral replication [10,11,19,22,79]. In this study, we enhance our understanding of the non-traditional monocyte-to-macrophage polarization induced by HCMV and the mechanisms involved in regulating the maturation machinery. Here, we show HCMV infection induces select M1 and M2 macrophage differentiation cell surface markers consistent with our previous transcriptome and chemokinome studies, confirming that HCMV stimulates a polarization of infected monocytes into macrophages possessing both M1 and M2 characteristics [24,25], which likely contributes to viral dissemination in a number of ways. Viral induction of M1 characteristics such as cellular activation, secretion of inflammatory cytokines, enhanced motility, adhesion to the endothelium, and transendothelial migration promote hematogenous spread of the virus from the blood into tissues [24,25]. Concomitantly, M2 properties likely control antiviral responses and evade the adaptive immune system [24,38].

The polarization of monocytes towards a hybrid M1/M2 phenotype occurs during infection by different HCMV strains, highlighting the evolutionary conservation of this viral dissemination strategy. In accord, different HCMV strains stimulate a persistent activation of Akt, which is necessary to drive the M1/M2 differentiation of infected monocytes after the 48-h viability gate. The mechanism by which Akt induces both M1 and M2 characteristics in infected monocytes is unclear as a role for Akt has been predominantly documented in M2 differentiation [63,65,80]. Multiple Akt isoforms, namely, Akt1, Akt2, and Akt3 could be involved in the non-traditional virus-driven differentiation of infected monocytes as Akt1 mediates M2 polarization and Akt2 mediates M1 polarization in mice [81]. Although we have observed robust Akt1 expression and low Akt2 expression in HCMV-infected monocytes (data not shown), MK-2206 potently inhibits both Akt1 and Akt2 allowing for the possibility that HCMV may use multiple Akt isoforms to induce the unique M1/M2 phenotype. Alternatively, we have shown Akt-dependent targets differ in HCMV-infected versus myeloid growth factor-treated monocytes [39]. Consequently, the distinct downstream targets of HCMV-activated Akt may be responsible for mediating the unique M1/M2 macrophage phenotype. Regardless of how Akt stimulates the hybrid phenotype, the mechanism by which Akt activity is regulated appears critical to determining the M1/M2 cell fate.

Akt activity is controlled by PI3K. We have previously shown that HCMV specifically utilizes the p110β isoform of PI3K to stimulate the survival of HCMV-infected monocytes [30]. Nevertheless, our results showed the p110δ isoform is largely responsible for the differentiation of HCMV-infected monocytes, suggesting different PI3K isoforms are required to mediate HCMV-induced monocyte survival versus differentiation. While both PI3K isoforms function to convert phosphatidylinositol 4,5-bisphosphate [PI(4,5)P_2_] into phosphatidylinositol 3,4,5-trisphosphate [PI(3,4,5)P_3_], each isoform has non-redundant functions in monocyte/macrophage biology [82]. While p110β is ubiquitously expressed, p110δ is selectively expressed in leukocytes and is the main isoform regulating Akt activity downstream of the M-CSF receptor, which stimulates monocyte differentiation towards M2 macrophages upon activation [82,83]. Our data here clearly demonstrate that p110δ is also involved in the acquisition of M1 characteristics during HCMV-induced monocyte differentiation. We previously showed SHIP1 to be selectively upregulated in HCMV-infected monocytes but not M-CSF treated monocytes, which led to the conversion of PI(3,4,5)P_3_ to PI(3,4)P_2_ and a distinct phosphorylation pattern on Akt [30]. Importantly, Akt activated during HCMV infection induced the expression of a unique subset of cellular pro-survival proteins, suggesting that SHIP1 could alter the biological output of p110δ. Indeed, SHIP1 inhibition prevented the upregulation of M1 markers consistent with previous studies [84], indicating the induction of SHIP1 during HCMV infection allows HCMV activation of p110δ to stimulate both M1 and M2 characteristics.

Caspases are key regulators of HCMV-induced monocyte-to-macrophage differentiation. Early inhibition of caspase 3 ensures the survival of infected monocytes past the 48-h viability checkpoint followed by a limited activation of caspase 3, which is required for the differentiation process. Akt has been implicated in conflicting reports to function as a positive and negative regulator of caspases during M-CSF-driven macrophage differentiation [44,58,69]. In agreement with these studies, we now show that Akt is used by the virus to temporally regulate caspase 3, whereby early Akt activity suppresses caspase 3 activation to promote survival [85], and that after the 48-h viability gate, Akt mediates the controlled activation of caspase 3 to drive differentiation [44]. How Akt plays a dual role in the temporal regulation of caspase 3 in HCMV-infected monocytes is unclear. The functional output of Akt is determined by its phosphorylation pattern [76,77,78], and we demonstrate a distinct Akt phosphorylation profile before and after the 48-h viability checkpoint in HCMV-infected monocytes. The different PI3K isoforms are known to impart unique phosphorylation patterns allowing for the isoforms to have distinct biological outcomes. Thus, perhaps p110β induces a phosphorylation pattern on Akt necessary to upregulate downstream pro-survival factors, such as Mcl-1 and HSP27 [39], which negatively control caspase 3 [27], whereas the pool of p110δ-activated Akt might allow for the triggering of a different set of targets, including those required for caspase 3 activation after the 48-h viability gate and macrophage differentiation. Regardless, our study illustrates that the precise control of Akt is essential to the temporal activation of caspase 3, and the subsequent virus-induced monocyte-to-macrophage differentiation process.

**Figure 6:**
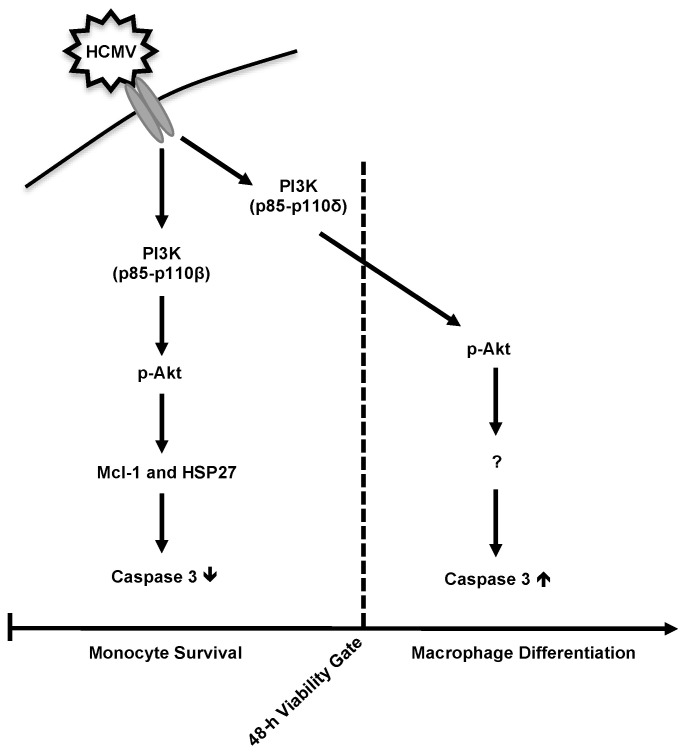
Potential model of HCMV-induced monocyte-to-macrophage differentiation. HCMV infection rapidly triggers PI3K-p110β signaling leading to a predominantly S473 phosphorylation of Akt and the upregulation of Mcl-1 and HSP27. Subsequently, the intrinsic biological programming of monocytes to activate caspase 3 is blocked by HCMV, allowing for infected monocytes to survive through the 48-h viability gate. Concomitant to PI3K-p110β activation, HCMV stimulates PI3K-p110δ leading to a distinct pool of activated Akt phosphorylated at S473 and T308 after 48 h. We hypothesize that this separate pool of Akt targets an unknown factor required for the controlled activation of caspase 3 after the 48-h viability gate in order to drive the unique M1/M2 macrophage differentiation of infected monocytes.

Overall, we demonstrate that the HCMV-induced monocyte differentiation process mediated by the temporal activation of caspase 3 is governed by the unique regulation of Akt signaling during infection (Figure 6). The induction of Akt prior to the 48-h viability gate inhibits caspase 3 activation and allows monocytes to bypass apoptosis initiation. After the 48-h viability gate, Akt activity stimulates the restricted activation of caspase 3 to drive monocyte differentiation. The ability of HCMV infection to recruit a distinct combination of Akt regulators, including different PI3K isoforms and SHIP1, allows for the unique biological output by HCMV-activated Akt leading to the unusual M1/M2 polarization needed for viral spread. Understanding the unique mechanisms by which HCMV drives monocyte-to-macrophage differentiation may provide new therapeutic interventions to prevent viral dissemination in high-risk immunocompromised patients.
